# A systematic review of interventions to enhance access to best practice primary health care for chronic disease management, prevention and episodic care

**DOI:** 10.1186/1472-6963-12-415

**Published:** 2012-11-21

**Authors:** Elizabeth Jean Comino, Gawaine Powell Davies, Yordanka Krastev, Marion Haas, Bettina Christl, John Furler, Anthony Raymont, Mark F Harris

**Affiliations:** 1 Centre for Primary Health Care and Equity, University of New South Wales, Sydney, NSW, 2052, Australia; 2 Centre for Health Economics Research and Evaluation, Faculty of Business, University of Technology, Sydney, Level 4, 645 Harris Street, Broadway, PO Box 123, Ultimo, NSW, 2007, Australia; 3 Perinatal and Women’s Mental Health Unit, St John of God Health Care & University of New South Wales, Sydney, Australia; 4 Primary Care Research Unit, Department of General Practice, University of Melbourne, 200 Berkeley St, Carlton, VIC, 3053, Australia; 5 Waitemata District Health Board, Waitakere Hospital, HealthWest Building, Private Bag 93115, Waitakere, 0650, NZ

**Keywords:** Primary health care, Family practice, Health services needs and demand, Health services accessibility, Diabetes mellitus, Papanicolaou test, After-hours care, Appointments and schedules, Continuity of patient care, English language

## Abstract

**Background:**

Although primary health care (PHC) is a key component of all health care systems, services are not always readily available, accessible or affordable. This systematic review examines effective strategies to enhance access to best practice processes of PHC in three domains: chronic disease management, prevention and episodic care.

**Methods:**

An extensive search of bibliographic data bases to identify peer and non-peer reviewed literature was undertaken. Identified papers were screened to identify and classify intervention studies that measured the impact of strategies (singly or in combination) on change in use or the reach of services in defined population groups (evaluated interventions).

**Results:**

The search identified 3,148 citations of which 121 were intervention studies and 75 were evaluated interventions. Evaluated interventions were found in all three domains: prevention (n = 45), episodic care (n = 19), and chronic disease management (n = 11). They were undertaken in a number of countries including Australia (n = 25), USA (n = 25), and UK (n = 15). Study quality was ranked as high (31% of studies), medium (61%) and low (8%). The 75 evaluated interventions tested a range of strategies either singly (n = 46 studies) or as a combination of two (n = 20) or more strategies (n = 9). Strategies targeted both health providers and patients and were categorised to five groups: practice re-organisation (n = 43 studies), patient support (n = 29), provision of new services (n = 19), workforce development (n = 11), and financial incentives (n = 9). Strategies varied by domain, reflecting the complexity of care needs and processes. Of the 75 evaluated interventions, 55 reported positive findings with interventions using a combination of strategies more likely to report positive results.

**Conclusions:**

This review suggests that multiple, linked strategies targeting different levels of the health care system are most likely to improve access to best practice PHC. The proposed changes in the structure of PHC in Australia may provide opportunities to investigate the factors that influence access to best practice PHC and to develop and implement effective, evidence based strategies to address these.

## Background

There is evidence that a strong primary health care (PHC) sector can improve individual and population health outcomes while limiting the cost of health service provision in general [1] and for particular health issues. For example, research has shown that early detection and active management of type 2 diabetes mellitus in primary health care reduces or defers onset of complications [2,3].

However, problems with availability, affordability or acceptability of services mean that PHC is not always readily accessible. Access can be compromised at many levels. At system level funding policies may make care unaffordable or planning may lead to a mal-distribution of services. At the service level billing policies may make services unaffordable to some, inflexible eligibility criteria or booking systems may reduce availability, and services may be culturally inappropriate, making them less acceptable to some patients [4,5]. Providers may not be sensitive to the health literacy, cultural background or service needs of some groups of patients, [4] and may have poor communication skills or discriminatory attitudes [6]. Barriers may also arise within the population, for example through societal, community or individual health attitudes, literacy and capacity for seeking and using health care. These provider and consumer factors interact, forming a dynamic system within which access is variously supported or hindered. This is well represented within an ecological model [7].

Apart from a few groups such as refugees and overseas workers, the Australian public has universal access to public hospitals and financial rebates for medical and some allied health care services under Medicare, Australia’s universal health insurance scheme. However there are still significant problems in accessing PHC, including an uneven distribution of services and patient co-payments for some services. In a 2009 international survey of primary care providers, only 36% of Australian general practitioners reported that most patients requesting same- or next-day appointment could get one, and only 50% of the practices had arrangements for patient’s after-hours care, placing Australia sixth out of seven advanced health care systems on this indicator [8,9].

Access to PHC is particularly important for conditions such as diabetes and for preventive care such as immunisation where PHC is known to be important and effective and there are well accepted management guidelines [10]. For chronic conditions continuity of care and proactive treatment of risk factors can improve health, prevent or delay complications and reduce cost such as hospitalisation.

In recent years a number of policy and practice initiatives in Australia have been introduced to improve access to PHC [11]. These include incentive payments to practitioners to relocate to areas of high need, funding for care planning for chronic conditions and provision of allied health care services in rural areas and an expansion of the range of PHC services that can be covered under Medicare [12]. While some of these interventions have been evaluated for their impact on access to PHC, there has been no systematic review of the effectiveness of strategies to improve access to primary health care. This paper reports the findings of a systematic review of the published peer and non-peer reviewed literature to identify effective interventions to improve access to best practice PHC.

## Methods

We conceptualised access as a dynamic balance between patient need and service provision, influenced by factors on both the health service/provider and patient/community sides of the equation and the relationship between them. We identified improvements in access through changes in patterns of service use. PHC was defined as first contact, community based health care services, largely but not exclusively based in general practice [13]. We focused on three domains of PHC which reflect different aspects of primary health care: episodic care, prevention, and chronic disease management. Within each domain we selected an area where guidelines are available with clear criteria for best practice processes of PHC: advanced access and after hours care for episodic care, Papanicolaou (Pap) tests for prevention and diabetes care for chronic disease management [10,14-16]. We recognised access to best practice PHC where there was evidence that one of these evidence based processes of care was received [10].

Electronic bibliographic databases (Medline, PubMed, Embase, APAIS Health, Cochrane, Epoch/ DARE) were searched to identify papers published between January 1999 and June 2009. The search terms related to ‘access to health care’ AND ‘PHC’ AND (‘diabetes’, ‘PAP testing’ OR ‘episodic care’) (Additional file 1: Appendix 1). Non-peer reviewed literature such as government reports were identified through relevant organisations or their websites and through consultation with key stakeholders (Additional file 2: Appendix 2). Additional citations were sought through the reference lists of relevant documents and hand searching targeted journals.

Studies were included if they were from Australia, New Zealand, the United Kingdom, Western Europe, USA or Canada; tested an intervention to improve access to one of our three domains of PHC; and measured access in terms of use of the relevant recommended processes of care. Evaluated interventions were assessed by one researcher (EJC) to score the methodological rigor and quality of evidence of the evaluated intervention studies, using the Quality Assessment Tool for Quantitative Studies, Effective Public Health Practice Project [17], and classified as low, medium or high.

Abstracts were screened for relevance. For those that met the selection criteria, the full paper was reviewed. Data were extracted using an extraction template developed for this study, and checked for completeness and accuracy by two members of the research team (YK and BK). Disagreements were discussed among the team and a consensus reached. Strategies were categorised as patient support, practice reorganisation, financial incentives, workforce development and provision of new services, recognising that some interventions addressed more than one of these categories. These were developed a priori and drew on previous research [18]. We then identified a subset of studies that measured the impact of interventions on change in use or the reach of services in defined population groups (evaluated interventions).

Simple descriptive statistics were employed. We noted the reported direction of change (positive (statistically significant increase in access), negative (non-significant increase in access), inconclusive (mixed, for example initial increase that was not sustained)), and related this to the type of intervention or strategy used, the setting and provider, characteristics of the target population and the health system level targeted. Evaluated interventions were also examined in regards to differential impacts for certain sub-populations and for cost-effectiveness.

Further details of the study protocol are available from the authors.

## Results

**Figure 1:**
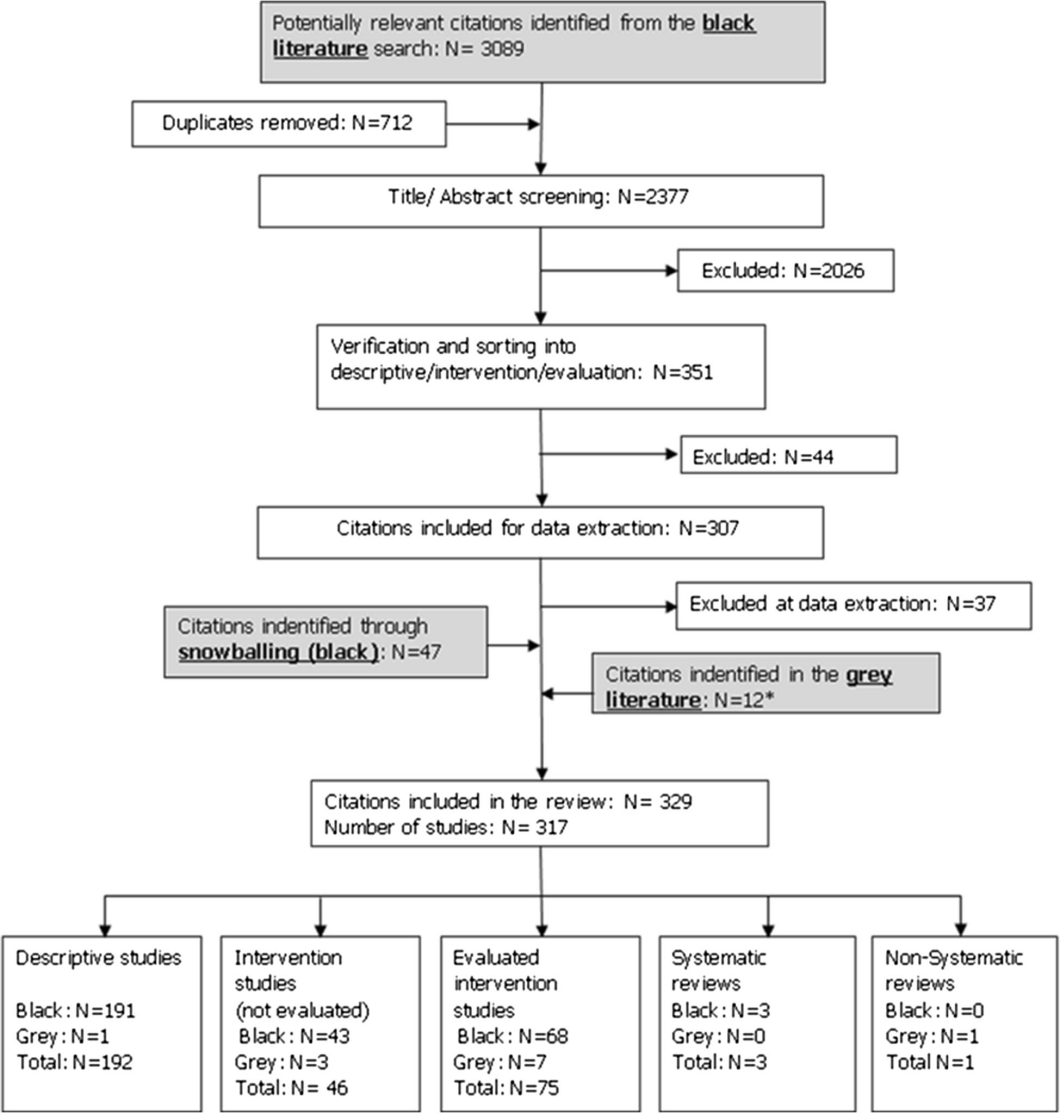
Flowchart for diabetes, PAP testing and episodic care literature searches.

The search identified 3,148 citations, of which 121 were intervention studies from one of three domains: prevention (Pap testing: n = 56), chronic disease domain (diabetes care: n = 38) and episodic care (n = 27) (Figure 1). Three were systematic reviews. Seventy five of the 121 intervention studies evaluated access in terms of change in processes of care and were included as evaluated interventions (Additional file 3: Appendix 3). The remaining 46 intervention studies measured change in access in terms of clinical outcomes such as HbA1c level or satisfaction with services rather than reporting change in access per se, and were excluded from further consideration.

Evaluated interventions were found in all three domains: prevention (n = 45), episodic care (n = 19), and chronic disease management (n = 11). They were undertaken in Australia (n = 25), the USA (n = 25), the UK (n = 15), Europe (n = 6), Canada (n = 3), and NZ (n = 1). The quality of 23 of the studies was rated as high (31%), 46 as medium (61%) and six as low (8%). The high quality evaluated interventions were all from the prevention domain, and were randomised control trials (n = 10) or longitudinal studies based on extract of testing records from large population based Pap testing registers (n = 13).

The main outcomes reported were changes in service use, provision of care processes such as evidence based screening, enhanced follow up or continuity of care, use of alternate services, and reduced waiting times. These varied by domain of care: 10 of 11 studies in chronic disease domain reported receipt of processes of care, 5 use of a service, and 2 reported change in follow up. 44 of 45 studies in preventive domain were about service use and completion of a Pap test, and 14 of 19 studies in episodic care domain were about use of services inclu-ding alternate services [7], and reduced waiting times [6].

The 75 evaluated interventions tested a range of 121 strategies either singly (n = 46 studies) or as a combi-nation of two (n = 19) or more strategies (n = 10). Stra-tegies targeted both health providers and patients and were categorised to five groups as follows: practice re-organisation (n = 46 studies), patient support (n = 29), provision of new services (n = 23), workforce development (n = 12), and financial incentives (n = 11) (Table 1). Examples of the sorts of strategies used are reported in Table 1. **Practice re**-**organisation** involved changing the way that care was offered e.g. establishing a condition specific clinic within the practice (n = 18 studies); introducing systems to support care e.g. patient registers and recall/reminder systems (n = 21); or external support for the practice e.g. from a practice support organisation or other resources (n = 7). **Patient support strategies** included reminder systems for patients and provi-ding education or educational material and information (n = 29 studies). **Provision of new services** included development of outreach services from existing clinics (n = 8 studies), or establishment of new services such as screening programs and walk in or after hour’s services (n = 15). **Workforce development** was concerned with improving provider skills and competencies or extending the providers’ roles e.g. nurses providing some screening or chronic disease management services (n = 12 studies). **Financial incentives** included both changes in fee structures and incentives to providers who met specified criteria (n = 9 studies).

**Table 1:** Summary of most common effective strategies used within evaluated intervention studies to enhance access to best practice process of PHC and indicating within each domain the types of strategies that were associated with report of positive or negative (italics) results

**Strategy type**	**Chronic disease: diabetes**	**Prevention: Pap test**	**Episodic care**
**Practice/ service reorganisation**			
Restructure of practice	·Multidisciplinary team care	·Greater focus on screening	·Changed appointment system
	·Disease specific clinic	·Enhanced risk assessment	·Same day appointments
	·Group attendance	·Nurse facilitated program	
Systems to support practice	·Personalised patient call/recall systems	·Office systems to identify	·Telephone triaging
	·Diabetes information and decision support systems	·compliance	·Reminders of appointments
		·Call/ recall/reminder systems	
External support for practice	·Diabetes register	·Establishment of condition specific registers	·*Doctor-operated after-hours telephone triage system*
		·Community awareness programs	
		·Population based programs	
**Patient support**			
	·Patient education /awareness raising	·Education / awareness programs personalised invitation to attend	·Telephone follow up of patients
	·Enhanced self-management	·Culturally appropriate materials and services	·Increased availability of same day appointments
		·Personalized invitations	
**New services**			
Outreach service	·Community based culturally specific clinic	·Outreach clinic,	·Outreach through home visits or phone
		·Home visit service	
New services to improve access	·Diabetes screening campaign	·Establishment of screening service	·Walk-in centres
		·Introduction of women’s health clinic	·After-hours care e.g. hospital based GP co-op
			·Nurse-led telephone triage
**Workforce development**			
	·Education of doctors about guideline-based diabetes care	·Education of doctors (e.g. use of screening guidelines)	
	·Enhanced role for other health providers	·Enhanced role for other health providers Training of lay health educators	
	·Education of other PHC providers, e.g. Nurses	·Culturally appropriate workforce	
**Financial incentives**			
	·Financial incentives for providers	·Reduce costs of screening	·Reduced cost/free service
	·Reduced cost for patients		
	·Change in funding rules		

**Figure 2:**
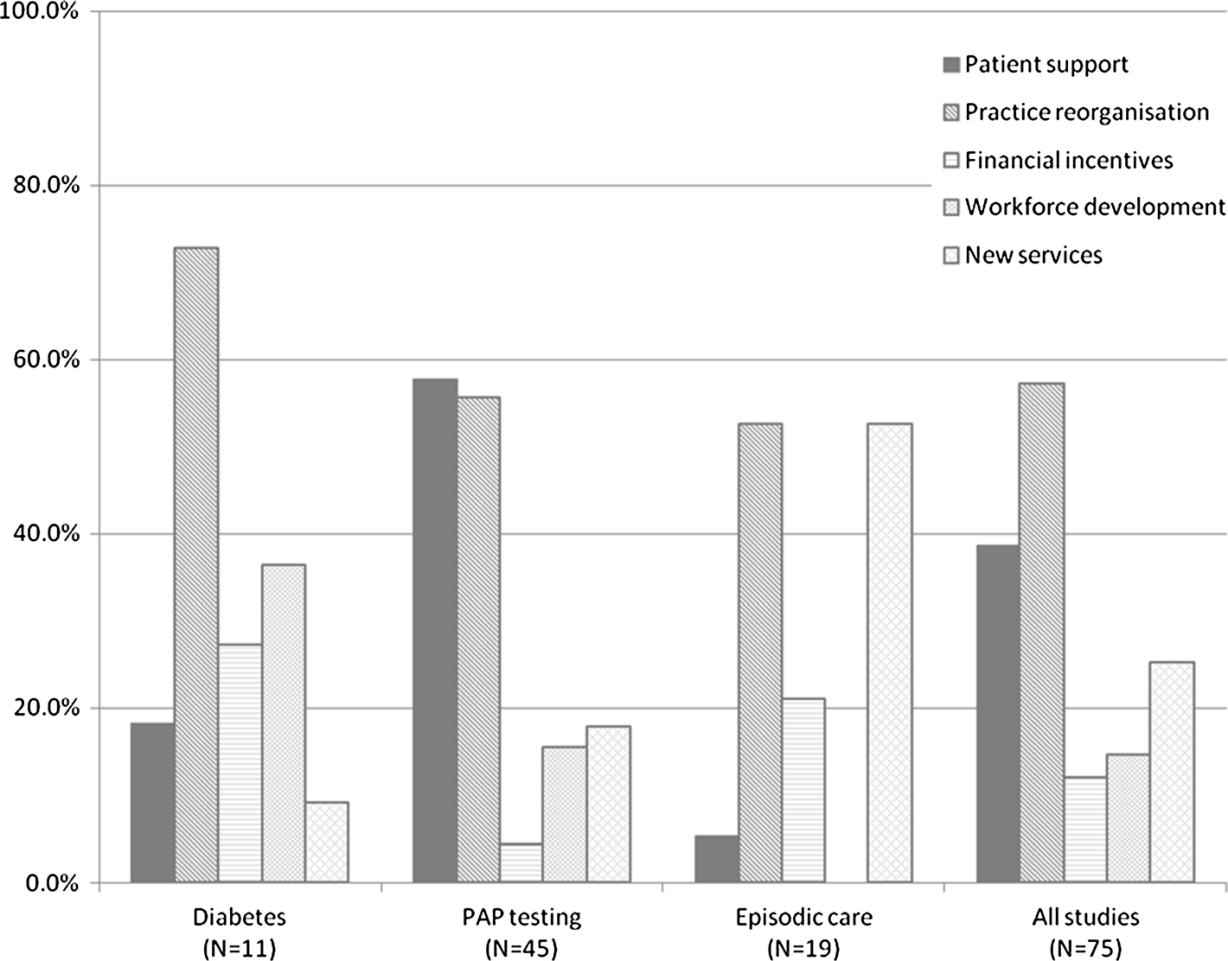
Frequency of strategies to enhance access to best practice PHC stratified by domain of care.

The choice of strategy types varied with the domain of care (Figure 2; Table 1). For example, access to chronic disease management (diabetes care) was improved through more regular review and monitoring, supported by practice reorganisation, systems to facilitate care, workforce development and financial incentives. For preventive care (Pap testing) the focus was on increasing patient attendance through better patient support and education, with the reminder systems and practice reorganisation needed to support this. Access to episodic care was addressed by establishing new services or practice reorganisation. The indicators of access also varied by domain: for diabetes care the use of services (n = 11) and receipt of particular processes of care (n = 9); for Pap testing the receipt of Pap testing (all studies); and for episodic care waiting time (n = 7), indications of continuity of care (n = 3) and use of services (n = 15).

The domains of care and types of strategies used varied also according to the country in which the study was conducted. Those from the USA (n = 25 studies) tended to be concerned with access to procedures, such as Pap testing (n = 20), improving access to diabetes care for specific population groups such as underinsured patients (n = 3), and improving efficiency and reducing costs of care within managed care organisations (n = 3). This contrasted with the UK (n = 16 studies), where national goals had been set for waiting times for GP appointments (n = 9). Most UK studies examined strategies to enhance access to episodic care, such as same day appointment, telephone triage, and out-of-hours services. Fewer papers addressed access to chronic disease management (diabetes care). The literature from Australia and New Zealand reflected the complex mix of public and private provision and funding of PHC and was concerned with all three domains of care.

Of the 75 evaluated interventions, 54 reported positive outcomes (a statistically significant increase in use of services or processes of care). Those using a combi-nation of strategies were more likely to report positive outcomes (Table 2). For example, a complex diabetes shared care intervention including participating practitioners, community based diabetes care nurses, agreed care protocols and structured communication strategies reported significant improvements in use of diabetes care, with more annual reviews and fewer patients defaulting for care [19]. For patients, public education campaigns had a demonstrated role in improving access to preventive care with the social media helping promote services, increase community expectations for screening programs such as Pap testing and increase screening rates [20,21]. Studies in the episodic care domain evaluated new services or booking systems to improve access to same day or afterhours care.

**Table 2:** Evaluated interventions by domain of care, number of strategies and positive outcomes

**Domain of care**	**Number of strategies used**			**Total**
	One (positive)*	**Two (positive)**	**Three or more (positive)**	
Diabetes	4 (3)	5 (4)	2 (2)	11 (9)
PAP testing	27 (15)	13 (11)	5 (5)	45 (31)
Episodic care	15 (12)	2 (1)	2 (2)	19 (15)
**Total**	**46 (30)**	**19 (15)**	**10 (9)**	**75 (55)**

* (positive): number of studies within group that reported positive change in access to best practice process of PHC.

Studies that reported inconclusive (n = 6 studies) or negative (n = 15 Studies) results provided several lessons. Some studies with mixed or inconclusive results could attribute lack of success to poor study design or lack of statistical power. A few studies demonstrated improved access initially and difficulties in maintaining these changes in access over time and particularly beyond the life of the project, for example at the conclusion of a public education campaign [22-24]. Initial and ongoing success depended in part on having a champion within the service to keep initiatives alive, implementation of sustainable practice systems to support care processes or on having policy or financial incentives for the change [25,26]. A few interventions and strategies reported unintended consequences: for example changed arrangements to increase availability of same day appointments (advanced access) in general practice made it harder to get planned appointments for ongoing chronic disease management with the consequence that poorer care was observed for those with chronic care needs [27]. Some studies showed differential uptake across population groups: for example culturally diverse groups, people who had never been screened, especially where the strategy was not tailored to the group in question [22,23]. Strategies involving new services also produced mixed results. For example, studies of the introduction of walk-in primary care clinics [28] and telephone triage [29] reported good use by patients although there was no reduction in use of pre-existing services. A new after-hours service did not show improvement in access to after-hours care due to patients’ misconceptions about how to access the new system [29].

We identified few studies that reviewed service costs. One study reported that fee reduction alone may not be effective if there are other barriers to attendance [29]. And finally only two studies evaluated the impact of policy [29,30] rather than more local, service oriented initiatives.

## Discussion

This paper reviewed intervention studies that evaluated strategies to enhance access to best practice processes of PHC in countries with well developed PHC systems although differing health insurance or financing mechanisms. The predominant topics and strategies in each country reflected problematic aspects of their health systems and current policies to address these. For example, in the UK the National Health System provides health care that is free at the point of care and thus is affordable, but sometimes a limited supply of available services exist. This was reflected in the focus of interventions on maximising access to same day episodic care through changed appointment systems within the limited services available. In the USA, by contrast, high overall costs and the absence of a system of universal health insurance cover are reflected in the predominance of interventions to improve affordability, availability and acceptability through establishment of systems of managed care and of reach to uninsured and marginalised groups. Evaluated interventions in the Australian setting also addressed all three access issues (affordability, availability, and acceptability), although interest was in improving coverage of universal system, thus reflecting the complexities of Australia’s mixed public-private system.

Interventions addressed aspects of access on both supply (service provision) and demand (seeking services), and at different levels: individual users and providers of services, communities and health service and health system level. They were most effective when they used a combination of strategies to improve access, often addressing both patient demand and service provision. For example, an effective intervention to improve access to diabetes education within general practices would include strategies which addressed workforce availability and skills, developed organisational systems to facilitate patient recall and education, ensured culturally appropriate ways of working, as well as appropriate reminders for patients, and addressed patient out of pocket expenses through additional alternate funding pathways such as practice incentive programs.

Most frequently, interventions targeted patients or service providers but not both, often focusing on clinical or practice systems to support more comprehensive care and appropriate follow up. This is consistent with current understanding of the importance of the practice environment for the delivery of best practice PHC [9]. It can also require support at different levels: for example the UK advanced access program was implemented locally but driven nationally and through regional organisations [31], and Australian initiatives to improve access to best practice chronic disease care at practice level have been supported by national funding initiatives and supported through the Divisions of General Practice network [32].

The types or intervention varied across the three domains of PHC that were studied. Multiple strategies were more frequently observed in chronic disease management domain, reflecting the complex arrangements needed to support consistent multi-disciplinary care. This is consistent with the Chronic Care Model [33], which identified several aspects of practice and care organisation that are needed to support an effective clinical partnership between primary care provider and patient. By contrast, single strategies were more frequently observed for preventive and episodic care (patient or population based screening registers or awareness programs and new appointment systems or new services respectively). This highlights the need for each access problem to be understood and addressed on its own terms. However it is also important to consider the impact of a change in access directed at one specific area on other types of service: generalist PHC practices provide a range of services. Strategies to improve access to one service such as same day episodic care (advanced access) can compromise access to other services such as planned chronic disease care [27,31].

Finally, most evaluated interventions studied were targeted at the whole population. There was evidence that some interventions that reported positive changes in access, failed to engage significant socio-demographic or cultural sub-populations. This highlights the close link between access and equity. Although there are mainstream problems of access to PHC services, the Inverse Care Law points out that some population groups make less use (or less effective use) of services in relation to their need [34,35]. Thus there can be a tension between improving access for all and targeting priority populations. Successfully addressing this requires good understanding of the access issue as well as implementation of complementary universal and targeted strategies. Further research in this area is needed.

Other than issues with study design, studies with negative or mixed results, while few, were informative. The issues relating to the success of interventions could be grouped into change drivers; duration and sustainability; reach accessibility and acceptability; and opportunism. Interventions that had wider support such as national policy or establishment of practice based systems were more likely to be successful and sustainable than those interventions that were local and dependant on a local champion or non-recurrent funding. Likewise, interventions that experienced differential uptake by population characteristics, may need to develop different strategies to address this. And finally opportunistic services such as screening located at sites where target populations assemble such as shopping or community cultural centres have potential for change although the literature suggested sustainability of these could be dependent on staffing or leadership.

Although this was a systematic review we did not attempt to undertake a meta-analysis due to the broad scope of evaluated intervention studies that were included. The primary focus of the review was on access to processes of care for the wider population. The research funding agreement excluded a specific focus on access to PHC for rural and remote areas where access to PHC is known to be a problem, although papers that were relevant to our search terms were not specifically excluded. There is a possibility of publication bias as most of the studies identified included positive results, studies were limited to English language, and most papers were focussed on primary medical care reflecting the structure of the health systems studied and lack of publications from other sectors. There was a dearth of evaluated interventions that explored the impact of interventions on differential changes in access for particular population groups. Further research is needed to identify those elements of complex interventions that are effective in enhancing access to primary health care.

## Conclusion

Ensuring that the population has access to quality PHC will continue to be an important goal for the Australian health system. This review suggests that multiple, linked strategies targeting different levels of the health care system are best placed to improve access to best practice PHC. We identified a number of elements of successful strategies and interventions to improve patient access to and use of processes of PHC. Overall these strategies targeted three areas:

1. System level change, service delivery policy, and financial incentives to provide incentives for wider dissemination of programs such as Pap testing;

2. Practice level reorganisation to provide better support and encouragement to effective multidisciplinary care, and develop practice systems to identify and follow up patients who require specific aspects of care, and

3. Community level programs to enhance engagement with the community such as outreach and other forms of service delivery that take services to patients, support patients through reminder systems to follow up on their health care needs, and public and social education programs to improve knowledge about appropriate services.

Interventions could be further enhanced or sustained through public policy and sustainable financial incentives and increased community awareness of services through public and targeted education programs. These strategies coincide with many of the recognised key elements of a well functioning PHC system. The proposed changes in the structure of PHC in Australia may provide opportunities to better understand the factors that influence access to best practice PHC and to develop and implement effective, evidence based strategies to address these.

## Competing interests

There are not competing interests.

## Authors’ contributions

EC led the research. She was responsible for all stages of the research including conceptualising the project, successfully applying for funding, employing the project staff, implementing the GPD this paper. PGPD was a senior member of the research group. He has contributed to all aspects of the implementation of the project, participated in the preparation of the report and conceptualisation and preparation of this paper. He was a member of the study advisory group. YK was employed as Research Fellow. She was responsible for the day to day implementation of the research, extraction and analysis of project data, preparation of the report with responsibility for specific sections, and preparation of this paper. MH was a senior member of the research group. She provided expert advice on economic aspects of the research. She was a member of the study advisory group and has made a significant contribution to the preparation of this paper. BC was employed as Project Officer. She was responsible, with the Research Fellow, for the day to day implementation of the research, extraction and analysis of project data, preparation of the report with responsibility for specific sections, and preparation of this paper. JF was a member of the research group. He was a member of the study advisory group and made a significant contribution to the conceptualisation and implementation of the project. He has contributed to all aspects of the implementation of the project and to the preparation of this paper. AR was a member of the research group. He was a member of the study advisory group and made a significant contribution to the conceptualisation and implementation of the project. He has contributed to all aspects of the implementation of the project and to the preparation of this paper. MFH was a senior member of the research group. He has contributed to all aspects of the implementation of the project, participated in the conceptualisation and implementation of the data extraction, preparation of the report and conceptualisation and preparation of this paper. He was a member of the study advisory group. All authors read and approved the final manuscript.

## Pre-publication history

The pre-publication history for this paper can be accessed here:

http://www.biomedcentral.com/1472-6963/12/415/prepub

## Supplementary Material

Additional file 1 **Appendix 1.** Search strategy for Chronic Disease papers (diabetes). Click here for file

Additional file 2 **Appendix 2.** Organisations and web pages searched for grey literature. Click here for file

Additional file 3 **Appendix 3.** Summary of evaluated intervention studies. Click here for file
