# Supplementary material for: A systematic review of interventions to enhance access to best practice primary health care for chronic disease management, prevention and episodic care
Source: BMC Health Serv Res. 2012 Nov 21;12:415. doi: 10.1186/1472-6963-12-415 (PMC3512489; doi:10.1186/1472-6963-12-415)
Supplement: Additional file 1 — Appendix 1. Search strategy for Chronic Disease papers (diabetes). [file 1472-6963-12-415-S1.doc]

### Appendix 1: Search strategy for Chronic Disease papers (diabetes)

| Medline (OvidSP) |
| --- |
| 1. exp Canada/ or exp United States/ or exp Europe/ or exp Great Britain/ or exp New Zealand/ or exp Australia/ 2. exp Primary health care/ or exp Ambulatory care/ or exp Community health services/ or exp Family practice/ or exp Family physician/s or exp personal health services/ 3. exp "Patient Acceptance of Health Care"/ or exp "Health Services Needs and Demand"/ or exp Health Services Accessibility/ or exp Health Manpower/ or exp Healthcare Disparities/ 4. exp diabetes mellitus, Type 2/ 5. 1 and 2 and 3 and 4 6. limit 5 to (abstracts and English language and humans and yr="1989 -Current" and "all adult (19 plus years)") |
| Embase (OvidSP) |
| 1. exp Europe/ or exp Canada/ or exp Australia/ or exp United States/ or exp New Zealand/ 2. exp primary health care/ or exp primary medical care/ or exp ambulatory care/ or exp community care/ or exp family medicine/ or exp general practice/ or exp general practitioner/ 3. exp health care access/ or exp health care need/ or exp health care availability/ or exp health care distribution/ or exp patient attitude/ or exp health care delivery/ or exp health manpower/ 4. exp self care/ or exp Self Efficacy/ or self-management support.mp. 5. exp Diabetes Mellitus 6. limit 5 to (English language and yr="1989 -Current" and (adult <18 to 64 years> or aged <65+ years>)) |
| CINAHL (EBSCO Host) |
| 1. (MH "Australia+") or (MH "New Zealand") or (MH "Canada+") or (MH "United States+") or (MH "Europe+") 2. (MH "Family Practice") or (MH "Ambulatory Care") or (MH "Primary Health Care") or (MH "Physicians, Family") or (MH "Community Health Services+") 3. (MH "Health Manpower+") or (MH "Health Services Needs and Demand+") or (MH "Health Services Accessibility+") or (MH "Health Care Delivery+") or (MH "Patient Attitudes") 4. MH Diabetes Mellitus 5. S1 and S2 and S3 and S4   Limiters : publication year from: 1998-2009; English Language; Age Groups: All Adult |
| PubMed |
| ((primary health care[MeSH Terms] OR ambulatory care[MeSH Terms] OR community health services[MeSH Terms] OR family practice[MeSH Terms] OR family physician[MeSH Terms] OR personal health services[MeSH Terms] AND (health services accessibility[MeSH Terms] OR patient acceptance of health care[MeSH Terms] OR health manpower[MeSH Terms] OR health care disparities[MeSH Terms]) AND (diabetes mellitus type 2 [MeSH]) AND (Canada[MeSH] OR United States[MeSH] OR Australia[MeSH] OR New Zealand[MeSH] OR Europe[MeSH]))  Limits: published in the last 10 years, English, All Adult: 19+ years |
| The Cochrane Library (Wiley Interscience) |
| 1. MeSH descriptor **Primary Health Care** explode all trees 2. MeSH descriptor **Ambulatory Care** explode all trees 3. MeSH descriptor **Community Health Services** explode all trees 4. MeSH descriptor **Family Practice** explode all trees 5. MeSH descriptor **Personal Health Services** explode all trees 6. MeSH descriptor **Health Services Accessibility** explode all trees 7. MeSH descriptor **Patient Acceptance of Health Care** explode all trees 8. MeSH descriptor **Health Manpower** explode all trees 9. (#1 OR #2 OR #3 OR #4 OR #5) 10. (#6 OR #7 OR #8) 11. MeSH descriptor **Diabetes Mellitus, Type 2** 12. (#9 AND #10 AND #11) 13. MeSH descriptor **Adult** explode all trees 14. (#12 AND #13), from 1989 to 2009 |
| Informit: Humanities & Social Sciences Collection, APAIS-Health, Health & Society |
| (((Australia) OR (New Zealand) OR (Canada) OR Europe OR USA) AND ((primary health care) OR (ambulatory care) OR (community health services) OR (family practice) OR (family physician)) AND ((health services accessibility) OR (health services needs and demands) OR (patient acceptance of health care) OR (health manpower) OR (health care disparities)) AND ((diabetes mellitus))  Limit to 1989-2009 |
