# Supplementary material for: A systematic review of interventions to enhance access to best practice primary health care for chronic disease management, prevention and episodic care
Source: BMC Health Serv Res. 2012 Nov 21;12:415. doi: 10.1186/1472-6963-12-415 (PMC3512489; doi:10.1186/1472-6963-12-415)
Supplement: Additional file 2 — Appendix 2. Organisations and web pages searched for grey literature. [file 1472-6963-12-415-S2.doc]

# Appendix 2: Organisations and web pages searched for grey literature

| **National** |
| --- |
| Australian Government, Department of Health and Aging [http://www.health.gov.au](http://www.health.gov.au/) |
| Royal Australian College of General Practitioners http://www.racgp.org.au/ |
| Medicare http://www.medicareaustralia.gov.au/provider/incentives/index.jsp |
| Australian Medical Association http://www.ama.com.au/ |
| National Health and Hospitals Reform Commission  http://www.nhhrc.org.au/ |
| Australian Primary Health Care Research Institute (APHCRI) <http://www.anu.edu.au/aphcri/> |
| Primary Health Care Research & Information Service (PHCRIS) <http://www.phcris.org.au/> |
| Australian General Practice Network (AGPN) <http://www.agpn.com.au/site/index.cfm> |
| Australian Primary Care Collaboratives (APCC) <http://www.apcc.org.au/> |
| Cancer screening http://www.cancerscreening.gov.au/ |
| Australian Institute of Health and Welfare (AIHW) <http://www.aihw.gov.au/publications/index.cfm/title/10646> |
| GP NSW [http://www.gpnsw.com.au](http://www.gpnsw.com.au/) |
| GP Access (Formerly Hunter Urban Division of General Practice) <http://www.gpaccess.com.au/> |
| Genera Practice Victoria <http://www.gpv.org.au/> |
| HealthOne <http://www.health.nsw.gov.au/initiatives/healthonensw/index.asp> |
| Fremantle Diabetes Study H<http://www.medicine.uwa.edu.au/go/fds/publications> |
| Australian Capital Territory Department of Health <http://www.health.act.gov.au/c/health?a=sp&pid=1227142283> |
| Australian Nursing Federation (AFN) <http://www.anf.org.au/> |
| Consumers’ Health Forum <http://www.chf.org.au/index.asp> |
| Diabetes Australia [http://www.diabetesaustralia.com.au](http://www.diabetesaustralia.com.au/) |
| School of Public Health and Community Medicine, UNSW  <http://www.sphcm.med.unsw.edu.au/sphcmweb.nsf/page/Australia> |
| Western Australia Department of Health http://www.health.wa.gov.au/home/ |
| Heart Foundation [http://www.heartfoundation.org.au](http://www.heartfoundation.org.au/) |
| National Asthma Council Australia <http://www.nationalasthma.org.au/> |
| NSW Health http://www.health.nsw.gov.au/ |
| **International** |
| New Zealand Ministry of Health <http://www.moh.govt.nz/> |
| Health Council of Canada [http://www.healthcouncilcanada.ca](http://www.healthcouncilcanada.ca/) |
| Statistics Canada [http://www.statcan.gc.ca](http://www.statcan.gc.ca/) |
| The Canadian Agency for Drugs and Technologies in Health <http://www.cadth.ca/index.php/en/cadth/products> |
| Centre-Urbanisation Culture Societe (INRS), Canada <http://www.ucs.inrs.ca/default.asp?p=> |
| Canadian Institute for Health Information (CIHI) [http://secure.cihi.ca](http://secure.cihi.ca/) |
| The Health Foundation, UK  <http://www.health.org.uk/publications/research_reports/cvd_prevention.html> |
| Bandolier (free database), UK <http://www.jr2.ox.ac.uk/bandolier> |
| Institute: Health and Life Sciences, UK <http://www.intute.ac.uk/healthandlifesciences/> |
| Department of Health, UK <http://www.dh.gov.uk/en/Publicationsandstatistics/Publications/PublicationsPolicyAndGuidance/DH_084970> |
| TRIP Database (Turning Research Into Practice), UK [http://www.tripdatabase.com](http://www.tripdatabase.com/) |
| Scottish Government Social Research [www.scotland.gov.uk/socialresearch](http://www.scotland.gov.uk/socialresearch) |
| NatPaCT, UK <http://www.natpact.nhs.uk/> |
| University of Liverpool - AMP (Access to mental health in primary care), UK  [www.liverpool.ac.uk/amp](../../../../%5C%5Cfiles1-med%5Csphcm%5CCPHCE%5Cprojects%5CRMO6722-%20APHCRI%20Access%5CAPHCRI%20reports%5Cfinal%20report%5CAppendices%5Cwww.liverpool.ac.uk%5Camp) |
| NIHR Service Delivery and Organisation programme  [http://www.sdo.nihr.ac.uk/accesstohealthholdingpage.html](http://www.sdo.nihr.ac.uk/accesstohealthholdingpage.html )H |
| King's Fund, UK <http://www.kingsfund.org.uk/> |
| The Nuffield Trust, UK <http://www.nuffieldtrust.org.uk/> |
| NLM Gateway, USA <http://gateway.nlm.nih.gov/gw/Cmd> |
| Academy Health, USA  <http://www.academyhealth.org/Publications/BriefList.cfm?navItemNumber=534> |
| The Commonwealth Fund, USA [http://www.commonwealthfund.org](http://www.commonwealthfund.org/) |
| New York Academy of Medicine's Grey Literature Collection, USA  <http://www.nyam.org/library/grey.shtml> |
| IQ Scientific Institute for Quality in Health Care,, Radboud University Nijmegen, The Netherlands http://www.iqhealthcare.nl/ContentFront/DefaultStart.aspx |
| European Centre for Social Welfare Policy and Research, Austria <http://www.euro.centre.org/detail.php?xml_id=866> |
| European Observatory on Health Systems and Policies, Belgium  <http://www.euro.who.int/observatory> |
| The Government and the Government Offices of Sweden <http://www.sweden.gov.se/> |
