# Supplementary material for: A systematic review of interventions to enhance access to best practice primary health care for chronic disease management, prevention and episodic care
Source: BMC Health Serv Res. 2012 Nov 21;12:415. doi: 10.1186/1472-6963-12-415 (PMC3512489; doi:10.1186/1472-6963-12-415)
Supplement: Additional file 3 — Appendix 3. Summary of evaluated intervention studies. [file 1472-6963-12-415-S3.doc]

# Appendix 3: Summary of evaluated intervention studies

| **1st Author** | **Year** | **Domain of Care** | **Country** | **Intervention summary** | **Strategy type** | **Study design** | **Quality Level *** | **Outcome summary** |
| --- | --- | --- | --- | --- | --- | --- | --- | --- |
| Agban, H. | 2008 | Diabetes | New Zealand | Reduced costs of care including Nationwide annual free diabetes review programme in PHC for all diabetes patients to improve diabetes care, changed prescribing rules for statins, and introduction of new guidelines | Fin | cohort | 2 | Care processes + |
| AIHW | 2009 | PAP test | Australia | Implementation and use of organised regular screening using the Pap test | Org3 | sequential cross-sectional study | 3 | Use of the service + |
| Anderson, R.M. | 2002 & 2003 | Diabetes | USA | Conducting community-based, culturally specific, eye disease screening clinics for urban African Americans with diabetes. Personalised telephone follow-up/ recall of patients. | Org2  Pat  Geo1 | cross-sectional & RCT | 2 | Use of the service + Follow-up rate + |
| Bird, J.A. | 1998 | PAP test | USA | A neighbourhood-based awareness raising intervention including small group educational sessions, educational material (in Vietnamese) distribution and promotional events. | Pat Work | Non-randomised controlled trial | 2 | Use of the service + |
| Blumenthal, D.S. | 2005 | PAP test | USA | Culturally sensitive community based cancer awareness program | Pat | Non-randomised controlled trial | 2 | Use of the service (no change) |
| Bowman, J. | 1995 | PAP test | Australia | Invitation letters for screening sent from provider or Regional Dept of Health | Pat | RCT | 3 | Use of the service + |
| Buehler, S.K. | 1997 | PAP test | Canada | Call/ recall system for attending cervical cancer screening | Org2 | RCT | 3 | Use of the service + |
| Burack, R.C. | 2003 | PAP test | USA | Adding PAP smear information to a mammography reminder system. | Org2 | RCT | 3 | Use of the service + |
| Burack, R.C. | 1998 | PAP test | USA | Patient and physician reminder for PAP screen | Org2 | RCT | 3 | Use of the service (no change) |
| Byles, J.E. | 1996 | PAP test | Australia | Reminder letters for women to attend screening | Org2 | Cluster randomised trial | 3 | Use of the service +- |
| Cecchini, S. | 1989 | PAP test | Italy | Physician education and prompts for screening | Work | Quasi-experimental study | 2 | Use of the service + |
| Chalder, M. | 2003 | Episodic care | UK | Implementation of NHS walk-in centres | Geo2 | time series | 2 | Use of other services + |
| Comino, E.J. | 2007 | Episodic care | Australia | Establishing GP cooperative after-hours service | Geo2  Fin | cross-sectional study | 2 | Use of the service + Use of other services + |
| Corkrey, R. | 2005 | PAP test | Australia | Brief advice Interactive Voice Response (IVR) cervical screening intervention | Pat | Cluster randomised trial | 2 | Use of the service + - |
| de Jonge, E. | 2008 | PAP test | Belgium | Registry-based invitation to non-attenders | Org2 | Quasi randomised trial | 2 | Use of the service + |
| Del Mar, C. | 1998 | PAP test | Australia | A culturally sensitive media campaign followed by personalised invitation letters in language of recipient | Pat | RCT | 3 | Use of the service (no change) |
| Dignan, M. B. | 1998 | PAP test | USA | Implementation of an culturally appropriate health education outreach program delivered by lay health educators. | Work Geo1 | Before - after study / interrupted time series | 3 | Use of the service + |
| Dixon, S. | 2005 | Episodic care | UK | Evaluation of implementation of advanced access | Org1 | Before - after study / interrupted time series | 2 | Waiting time +  Use of the service + |
| Dunt, D. | 2002 | Episodic care | Australia | Telephone triage of after hours calls | Geo2 | Before - after study | 2 | Use of the service + |
| Dunt, D. | 2002 | Episodic care | Australia | Telephone triage service (Health Connect) | Geo2 | Before - after study | 2 | Use of the service (no change) Use of other services (no change) |
| Dunt, D. | 2002 | Episodic care | Australia | Telephone triage system (After Hours Doctor) | Org1  Fin | Before - after study | 2 | Use of the service - |
| Dunt, D. | 2006 | Episodic care | Australia | Combination of after hours services | Org1  Geo2  Fin | Before - after study | 2 | Use of the service + |
| Dunt, D. | 2002 | Episodic care | Australia | Telephone triage service (HealthDirect) | Geo2 | Before - after study | 2 | Use of the service (no change)  Use of other services (no change) |
| Edwards, R. | 2003 | Diabetes | UK | Introduction of a diabetes information system to support a guideline-based integrated diabetes care program | Org2  Work | sequential cross-sectional study | 1 | Care processes + |
| Fairhurst, K. | 2008 | Episodic care | UK | Text message reminder of appointments for patients who have missed appointments in the past. | Org2 | RCT | 2 | Use of the service + |
| Fernandez, M.E. | 2009 | PAP test | USA | Breast and cervical cancer education program provided by lay health workers | Pat | Before - after study / interrupted time series | 2 | Use of the service + |
| Gill, J.M. | 1998 | PAP test | USA | Provider training, involvement of nursing staff and new office flow chart system | Work Org2 | cross-sectional study | 2 | Use of the service + |
| Gottlieb, N.H. | 2001 | PAP test | USA | Put Prevention into Practice (PPIP) Program: office systems change for routine delivery of clinical preventive services | Pat Work Org3 | Before - after study / interrupted time series | 2 | Use of the service + |
| Grewal, S. | 2004 | PAP test | Canada | Establishment of culturally appropriate PAP test clinics | Geo1  Geo2 Pat | Before - after study / interrupted time series | 1 | Use of the service - |
| Hancock, L. | 2001 | PAP test | Australia | A community action program to raise awareness for cervical cancer and screening | Work Pat Geo1 | cluster randomised controlled trial | 3 | Use of the service + |
| Harris, M.F. | 2002 | Diabetes | Australia | A computerised diabetes register maintained by the Division; Division mails reminders to GPs for patient recall at various time intervals. | Org1  Org3 | Before - after study / interrupted time series | 2 | Use of the service + Care processes + |
| Hersberger, K.E. | 2006 | Diabetes | Switzerland | Free-of-charge diabetes screening campaign in community pharmacies. | Work  Pat  Fin  Geo2 | Before - after study / interrupted time series | 1 | Use of the service + Care processes + |
| Hirst, S. | 1990 | PAP test | Australia | A regional multifaceted screening campaign | Pat Org1 | Before - after study / interrupted time series | 3 | Use of the service + |
| Hogg, W. | 2008 | PAP test | Canada | Nurse facilitator practice support program for preventive health care | Org3 | cluster randomised trial (matched pairs) | 2 | Use of the service (no change) |
| Hunt, J.M. | 1998 | PAP test | Australia | Introduction of a women's clinic at an Aboriginal community health service and trialling 3 invitation mode. | Geo2 Pat Org2 Fin | RCT | 3 | Use of the service (+) |
| Kreuter, M. | 1996 | PAP test | USA | Enhanced health risk assessment and feedback to patient | Org1 | RCT | 2 | Use of the service (no change) |
| Lancaster, G. | 1992 | PAP test | UK | Combined invitation letter for cervical an breast cancer screening | Org2 | RCT | 3 | Use of the service + |
| Lantz, P. | 1995 | PAP test | USA | Invitation letters for screening test combined with follow up telephone counselling | Org2 Pat | Quasi experimental study | 3 | Use of the service + |
| Larkey, L. | 2006 | PAP test | USA | Community health adviser outreach program to educate women about cancer | Geo1  Pat | Before - after study/interrupted time series | 1 | Use of the service (no change) |
| Marcus, A.C. | 1992 | PAP test | USA | Comparison of three interventions to encourage women to attend to screening | Org2 Pat Fin | RCT | 2 | Return rates (+) |
| McAlister, A. L. | 1995 | PAP test | USA | Culturally sensitive health promotion campaign at community level | Pat | Non-randomised controlled trial | 2 | Use of the service (no change) |
| McAvoy, B.R. | 1991 | PAP test | UK | Multilingual health education | Geo1 Pat | RCT | 3 | Use of the service + |
| Mehrotra, A. | 2008 | Episodic care | USA | Implementation of advanced, open, or same day access | Org1 | Case series | 1 | Waiting time +  Use of the service (no change) |
| Mock, J. | 2007 | PAP test | USA | Lay health worker outreach and media-based education for promoting cervical cancer | Geo1  Work  Pat | RCT | 2 | Use of the service + |
| Morrell, S. | 2005 | PAP test | Australia | Personally addressed reminder letter from the state PAP Test Register for women who were under screened. | Org3 | RCT | 3 | Use of the service + |
| Munro, J. | 2000 | Episodic care | UK | Introduction of NHS Direct telephone triage service | Geo2 | Before - after study / interrupted time series | 2 | Use of other services + |
| Newell, S.A. | 2002 | PAP test | Australia | Personal health book records for patients | Pat | cluster randomised controlled trial | 3 | Use of the service (no change) |
| Nygard, J.F. | 2002 | PAP test | Norway | A population based, nationwide, cervical cancer screening programme for women of 25 to 69 | Pat Org3 | Before - after study / interrupted time series | 2 | Use of the service + - |
| O'Cathain, A. | 2007 | Episodic care | UK | Introduction of NHS direct and walk-in clinics. | Geo2 | cross-sectional study | 2 | Use of other services (no change) |
| Ornstein, S. M. | 1991 | PAP test | USA | Computer generated physician and patient reminders for preventive services | Org2 | RCT | 2 | Use of the service +- |
| Panaretto, K. S. | 2006 | PAP test | Australia | A community based health promotion program (Well Women's Check) to raise awareness for PAP testing combined with reminder letters | Pat Org3 | cohort study | 2 | Use of the service + |
| Pickin, D.M. | 2004 | Episodic care | UK | Set of interventions to improve access for patients e.g. Telephone consultations for follow-up appointments or reduced proportion of appointments bookable in advance. | Org1 | cross-sectional study | 2 | Waiting time +  Use of the service + |
| Pierce, M. | 1989 | PAP test | UK | Provider prompts versus invitation letters | Org2 | RCT | 2 | Use of the service + |
| Pritchard, D. A. | 1995 | PAP test | Australia | Different recruitment strategies for cervical screening in a general practice | Org2 | RCT | 3 | Use of the service + |
| Rimer, B.K. | 1999 | PAP test | USA | Computerised screening prompts for physicians combined with tailored print communication (TPC) and tailored telephone counselling for their patients | Org2 Pat | RCT | 3 | Use of the service + |
| Robson, J. | 1989 | PAP test | UK | Risk factor assessment through health promotion nurse at practice | Org1 | RCT | 3 | Use of the service+ |
| Roetzheim, R. G | 2004 | PAP test | USA | Office monitoring and recall system for cancer screening | Org2 | Cluster randomised trial | 3 | Use of the service + |
| Rohrer, J.E. | 2007 | Episodic care | USA | Same-day scheduling (advanced access or open access) to increase access to primary care provider | Org1 | cross-sectional study | 2 | Use of the service + |
| Salisbury, C. | 2007 | Episodic care | UK | Advanced access processes implemented in general practices | Org1 | Before - after study / interrupted time series | 2 | Waiting time +  Continuity of care (no change) |
| Salisbury, C. | 2002 | Episodic care | UK | Implementation of walk-in centres | Geo2 | time series | 2 | Waiting time + Use of the service + |
| Scott, A. | 2009 | Diabetes | Australia | Nationwide introduction of the Practice Incentive Program (PIP) to encourage higher quality of care in diabetes, asthma, mental health, and cervical screening. | Fin | cohort study | 2 | Care processes+ |
| Shelley, J.M | 1991 | PAP test | Australia | A state-wide mass media campaign on cervical cancer screening | Pat | Before - after study / interrupted time series | 1 | Use of the service + |
| Si , D.(2006) & Bailie, R.S.(2004) | 2004 | Diabetes | Australia | Supporting health centre staff for use of guidelines in remote Aboriginal communities. Promotion of the employment of AHW and their involvement in diabetes care. | Org2  Work | cohort study & time series | 2 | Care processes +  Use of the service + - |
| Smith, S. | 2004 | Diabetes | Ireland | Implementation of a structured diabetes shared care service through practice nurses and community diabetes specialist nurse. | Org1  Work | Cluster randomised trial | 2 | Use of the service + Retention + Care processes + |
| Solberg, L. | 2004 | Episodic care | USA | Evaluation of the implementation of advanced (open) access | Org1 | cohort study (retrospective) | 2 | Use of the service + Continuity of care + |
| Sturmberg, J.P. | 1999 | Diabetes | Australia | General practice diabetes clinics conducted through division dietitian and diabetes educator | org1 | Before - after study / interrupted time series | 2 | Care processes + |
| Subramanian, U. | 2009 | Diabetes | USA | Implementation of open access appointment system. | Org1 | Quasi-experimental study | 2 | Care processes - |
| Taylor, V.M. | 2002 | PAP test | USA & Canada | Trial of different screening recruitment strategies: outreach vs. Direct mailing | Pat Geo1 | RCT | 3 | Use of the service + |
| The Scottish Government | 2008 | Episodic care | UK | Implementation of advanced access | Org1 | Case studies | 2 | Waiting time +  Continuity of care + |
| The Scottish Government | 2008a | Diabetes | UK | Implementation of diabetes management strategies | Org1  Org2 | Case studies | 2 | Care processes + |
| Valanis, B.G. | 2003 & 2002 | PAP test | USA | Trial of different recruitment strategies to cancer screening: outreach vs. at point-of service reinforcement vs. Combined | Org2 Org1 | RCT | 3 | Use of the service + Time to service+ |
| van Uden, C.J. | 2005 | Episodic care | Netherland | Primary care physician cooperative to provide after-hours care. | Geo2  Pat  Fin | Before - after study / interrupted time series | 2 | Use of the service+  Use of other services + |
| Ward, J.E. | 1991 | PAP test | Australia | Two different strategies for GPs to encourage women to have PAP smear | Pat | Non-randomised controlled trial | 2 | Use of the service (no change) |
| Yancey, A.K. | 1995 | PAP test | USA | Culturally sensitive health education videos played in waiting room | Pat | Quasi-experimental study | 3 | Use of the service + |
| Young, M. | 1990 | PAP test | Australia | Media campaign and community based promotion | Geo1 Pat | Before - after study/interrupted time series | 2 | Use of the service + |

Pat = Patient support; Org1 = Reorganisation of practice; Org2= systems to support practice; Org3=External support for practice; Fin = Financial support; Work = workforce development; Geo1 = Geographical intervention (outreach service); Geo2 = Geographical intervention (other services to improve access)

* Quality of study [78]: 3 – high; 2 – medium; 1 – low.

# References:
